# Open source tools: an evaluation for digital forensic investigations

**DOI:** 10.3389/frma.2026.1790333

**Published:** 2026-02-19

**Authors:** Indra Abeysekera

**Affiliations:** Ton Duc Thang University, Ho Chi Minh City, Vietnam

**Keywords:** digital forensic tools, forensic investigation, open source tools, proprietary tools, traces

## 1 Introduction

Forensic science has historically lacked a uniform definition, which has often blurred the distinction between trusting the investigator and trusting a scientific method, thereby compromising the legal acceptance of forensic evidence (Koehler et al., 2023). In response, the 23rd meeting of the International Association of Forensic Science proposed a definition, based on seven foundational principles, now known as the Sydney Declaration. It defined forensic science as a “case-based (or multi-case-based) research-oriented, science-based endeavor to study traces—the remnants of past activities (such as an individual's presence and actions)—through their detection, recognition, recovery, examination and interpretation to understand anomalous events of public interest (e.g., crimes, security incidents)” (Roux et al., 2022, p. 2).

The seven principles noted in the definition were that: (1) traces produced through activities is the foundation for vector information, (2) investigating a scene must be conducted with scientific expertise with a scientific and diagnostic lens, (3) investigating a forensic scene is case-based relying on investigative methodology and logical reasoning, (4) assess findings acknowledging that time is asymmetrical, (5) be prepared being called to deal with a range of uncertainties, (6) has multiple purposes for conducting an investigation and can make multiple contributions, and (7) the context in which investigation conducted is essential to derive meaning (Roux et al., 2022).

Digital forensics is part of forensic science but has distinct nuances, focusing on cybercrime prevention, detection, and prosecution. Digital traces are more varied and can be found across different platforms over the temporal span of the investigations. Hence, digital forensics requires more sophisticated forensic tools that must be continually updated and improved to capture the breadth and depth of traces found in current technologies and those being developed and released to the market at an exponential rate (Klasén et al., 2024).

Given the expectation of multiple locations of traces and multiple platforms on which traces can be found during a launched digital forensic investigation, the investigation presents challenges, including the cost of conducting it, the accuracy of findings, and the depth and breadth of investigative methodologies needed to uncover the truth. The paper aims to evaluate whether open source tools should be considered only as a means of assistance and to determine whether open source digital tools alone are sufficient for forensic digital investigations (Overill et al., 2009).

## 2 Argument

### 2.1 Importance of digital forensic tools

The digital tools are worthy of such examination for seven reasons: First, they can analyze large, heterogeneous data sets. Second, they can help detect relevant and accurate data. Third, they enable data processing for interpretation. Fourth, they help in understanding the data, thereby aiding the investigation. Fifth, tools can analyze results visually. Sixth, they can evaluate and report findings. Seventh, digital tools positively influence the forensic loop, as their predictive capabilities enable new ways of analyzing heterogeneous data for investigation, with such predictive accuracy ranges aiding in refining investigations (Klasén et al., 2024).

### 2.2 Forensic tools classification

There are various ways to classify forensic tools: (1) Domains (operating system, disk and file system, live memory, web, email, network, and multimedia). (2) Licensing (open source, freeware, and proprietary). (3) Interacting tools (graphical user interface and command line), (4) platform (Windows, Linux, macOS, Android OS, and DOS), and (5) image file format supported [raw image, split raw image, and disk image (Javed et al., 2022)]. These classifications are interactive in forensic tools, as each, along with others, influences their limitations and expansiveness.

There are numerous open source digital forensic tools; among them, 11 established tools are: (1) Autopsy, (2) Prodsolver, (3) FTK, (4) OSForensics, (5)Network Miner, (6) LogRhythm, (7) NikSun, (8) Nmap, (9) XPLiCO, (10) Volatility Framework, and (11) Rekall.

Determining whether open source toolkits can be used exclusively in forensic investigations requires comparisons, which include both freeware and proprietary software. A Freeware digital forensic toolkit is Redline. The proprietary software includes: (1) Belkasoft, (2) XWays, (3) Encase, (4) Magnet, (5) WS, (6) F-Response, (7) Plixer, and (8) Magnet Axiom.

### 2.3 Digital traces

The digital traces serve as knowledge objects that assist investigators (Sunde, 2022). Ninety per cent of all recorded crimes have a digital association (NPCC, 2020). A digital forensic investigation commences when a cybercrime is first reported or when it is accidentally discovered to have occurred, is occurring, or is about to occur. It is likely that the investigation begins with little direct evidence of traceable activities and instead relies on circumstantial evidence. Digital activities involve interactions with subjects or objects, leaving digital traces consistent with Locard's Exchange Principle. They leave investigators with the challenge of establishing digital activity or source traces, and vice versa (Horsman, 2024).

Traces of past activity leading to sources, and sources leading to past activity related to the investigation, may be distributed across multiple locations or domains in technologies such as a computer. They can be found in the operating system, disk and file systems, live memory, on the web, in email, on networks, and in multimedia. Digital tools can play a crucial assisting role (Javed et al., 2022).

In operating systems, suspicious files and activity can be traced by matching hashes, comparing signatures, analyzing memory use, and examining binary data. The sources of traces are commonly found in recycle bins, event logs, link files that maintain metadata, prefetch files that speed up application processes, timelines, telemetry, AmCache, ShimCache, retrieved passwords, and the System Resource Usage Monitor (SRUM) (Javed et al., 2022).

Traces in the file and disks domain require examining the file system explorer, slack space not yet utilized by files, viewing the hex values of files as they exist in the form of bits, carving deleted files to recover their metadata, back copies of Windows files, and the Windows registry that records user activities, analyzing malware, analyzing hibernation files, and analyzing data stored in Redundant Array of Independent Disks (RAID) (Javed et al., 2022).

Traces in live memory (Random Access Memory, RAM) domain require examining the command-line interface and the graphical user interface, carving memory dumps, and recovering data that has been deleted, corrupted, or hidden in memory. Unused (slack) space in data structures can contain traces of previous or existing data, enable live analyses on technologies such as computers that are still connected to power, or image RAM because it is too large; otherwise, retrieval takes a long time (Javed et al., 2022).

Finding traces on the web requires examining the bookmarks, browser history, downloads, search queries, cookies, and cache. Traces in emails are examined in the email headers, mailbox, and email type (Javed et al., 2022).

Traces in networks are obtained by capturing data packets in network flows, scanning ports, collecting logs maintained by network devices, and analyzing threats that prevent and detect intrusions. Traces can be found in multimedia to authenticate images, detect objects in images, and reconstruct compromised or distorted images (Javed et al., 2022).

## 3 Hypotheses

Technology encompasses a wide range of devices; however, for this paper, the criteria include computer systems, networks, and multimedia, excluding mobile devices, the Internet of Things, and cloud-based platforms. The criteria were influenced by the output data reported in the journal paper (Javed et al., 2022). Those data were examined in this study by stating the null hypothesis:

H_0_: Forensic investigations should only be conducted using open source tools.

**Figure 1:**
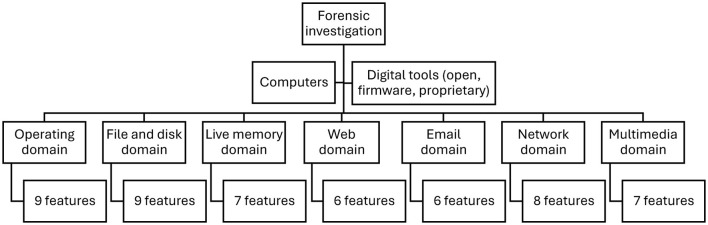
Forensic investigation domains and features in a computer.

The hypothesis is qualitatively tested in two dimensions: domain systems and features within these domain systems. Figure 1 presents the framework used in this study to qualitatively examine the hypothesis. There are seven domain systems covered in a forensic investigation of a computer. It is crucial to focus on domain systems because each domain has its own set of investigative models. The first sub-hypothesis tested is H_0A_. The default position is that open source tools are sufficient for comprehensive forensic investigations of computers.

H_0A_ Forensic investigations of all domain systems should only be conducted using open source tools.

Figure 1 shows the number of features or functionalities representing each domain system. The features of each domain system are unique (Al-Dhaqm et al., 2021). However, a counterargument is that, in computers, domain- and feature-level differences are less conspicuous than in mobile systems and the Internet of Things. The open source tools are therefore sufficiently comprehensive for forensic investigations of features within each domain system on a computer. The second sub-hypothesis tested is H_0B_, which is stated as follows.

H_0B_ Forensic investigations of all domain features contained in domain systems should only be conducted using open source tools.

To test the hypothesis, this study used Javed et al.'s (2022) study as a secondary data source for qualitative analysis. The premise for the findings is established by comparing open source, freeware, and proprietary digital tools, each scored on a 0–100 scale. Open source software is characterized by transparent source code that allows users to modify it, whereas freeware can be used freely. In contrast, proprietary tools are offered for a fee, and in both freeware and proprietary tools, source code is not disclosed to users (Ambhire and Meshram, 2021). The basis of the conclusion is that traces and sources enable us to uncover facts and recreate the truth (Carvey and Althede, 2011).

## 4 Results

### 4.1 Comparing domains

This section reports the findings of the testing of H_0A._ Javed et al. (2022) reported scoring from 0 to 100, indicating whether a given tool supports the features identified for each domain. Instead, this paper normalized the nominal scores reported in Javed et al.'s (2022) study by converting them to percentages. Table 1 summarizes the output scores by domain system (operating, file and disk, live memory, web, and email), as shown in Panel A using open source tools, and Panel B using freeware (free) and proprietary (prop) tools. The network is shown in Panel C, and multimedia in Panel D, as they are distinct domain systems.

**Table 1:** Domain system digital forensic tools.

***Panel A*****. Open source (except network and multimedia domain systems)**.							
**Domain system**	**Autopsy**	**PD**	**FTK**	**Volatility framework**	**Rekall**	**FR**	**WS web services**
**Tool type**	**Open**	**Open**	**Open**	**Open**	**Open**	**Open**	**Open**
Operating	66	55	100				
File and disk	88	66	100				
Live memory				71	85	57	42
Web	83	0	100				
Email	66	50	83				
***Panel B*****. Freeware and proprietary (except network and multimedia domain systems)**.					
**Domain system**	**Redline**	**BEC**	**OSF**	**Xways**	**Encase**
**Tool type**	**Free**	**Prop**	**Prop**	**Prop**	**Prop**
Operating	55	88	88	66	66
File and disk	44	100	88	88	77
Live memory	14	85			71
Web	83		83	66	83
Email		83	83	66	83
***Panel C*****. Network domain system**.						
**Domain system**	**Network miner**	**Nmap**	**Xplico**	**LogRhythm**	**Plixer**	**NIKSUN**
**Type**	**Open**	**Open**	**Open**	**Prop**	**Prop**	**Prop**
Network	77	77	11	77	66	55
***Panel D*****. Multimedia domain system**.						
**Domain system**	**IntaForensics**	**Amped**	**Cognitech**	**FMDES**	**AMR**	**Avdetective**
**Type**	**Prop**	**Prop**	**Prop**	**Prop**	**Prop**	**Prop**
Multimedia	85	71	85	71	41	71

The results in Panel A indicate that no single open source solution is effective across all listed domain systems. FTK, an open source tool, scored 100% in operating, file-and-disk, and web domain systems. However, it was not functional in the live memory domain and scored 83% on the email system. Panel B results show that only one proprietary solution, Encase, was effective across all domain systems; however, it scored below 100% on them. Other proprietary solutions did not cover all domain systems.

Panel C focuses on the network domain system, while Panel D addresses the multimedia domain system. They required specialized tools. In network domain system forensic investigations, digital forensic tools comprised both open source and proprietary software, but none scored 100%. All investigative tools used in multimedia domain system forensic investigations were proprietary; none scored 100%.

Table 1′s findings suggest that open source digital forensic tools alone are ineffective, as none scored 100% across all domain systems. It is risky to disregard or select only certain domain systems of an investigation at the conceptualization stage, because when an investigation commences with circumstantial evidence, traces may exist across all systems. For instance, in occupational fraud, 52% of leads come from employees, 21% from customers, and 11% from vendors, which may begin as circumstantial evidence (ACFE, 2024). Hence, a more effective approach is to have a portfolio of tools to cover all domains in an investigation. Relying solely on open source tools can be risky, as they do not comprehensively cover potential trace examination across all domains for forensic investigations. Based on secondary data analyzed in this study, multimedia is a rapidly growing domain that currently relies on proprietary software tools, with open source tools yet to exist.

### 4.2 Comparing features in the domain

**Figure 2:**
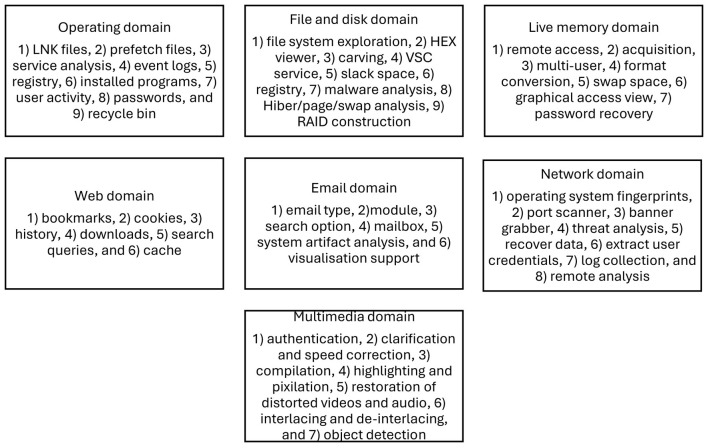
Features in domains.

This section reports the findings of the testing of H_0B._ Figure 2 shows the features in the domain systems reported by Javed et al. (2022), which were used to analyze and report in this study.

Table 2 presents the number of features in each domain system considered for digital forensic investigation. The analysis considered seven domain systems: operating, file and disk, live memory, web, email, network, and multimedia for feature selection (Javed et al., 2022).

**Table 2:** Features covered by the domain system digital forensic tools.

**Panel A. Open source. (except network and multimedia domains)**.								
**Domain system**	**# features**	**Autopsy**	**PD**	**FTK**	**Volatility framework**	**Rekall**	**F-Response**	**WS Web Services**
**Tool type**		**Open**	**Open**	**Open**	**Open**	**Open**	**Open**	**Open**
Operating	9	6	5	9				
File and disk	9	8	6	9	7			
Live memory	7				5	6	4	
Web	6	5	0	6				
Email	6	4	3	5				
**Panel B. Freeware and proprietary (except network and multimedia domains)**.						
**Domain system**	**# features**	**Redline**	**BEC**	**OSF**	**Xways**	**Encase**
**Tool type**		**Free**	**Prop**	**Prop**	**Prop**	**Prop**
Operating	9	6	8	8	6	6
File and disk	9	4	9	8	8	7
Live memory	7	1	6			5
Web	6	5		5	4	5
Email	6		5	5	4	5
**Panel C. Network domain**.							
	**# features**	**Network miner**	**Nmap**	**Xplico**	**LogRhythm**	**Plixer**	**NIKSUN**
**Type**		**Open**	**Open**	**Open**	**Prop**	**Prop**	**Prop**
Network	8	7	7	1	7	6	5
**Panel D. Multimedia**.							
	**# features**	**IntaForensics**	**Amped**	**Cognitech**	**FMDES**	**AMR**	**Avdetective**
**Type**		**Prop**	**Prop**	**Prop**	**Prop**	**Prop**	**Prop**
Multimedia	7	6	5	6	5	3	5

The findings reported in Table 2 suggest that FTK, an open source software, covers all forensic investigation features across the domain systems (operating = 9/9, file and disk = 9/9, web = 6/6), except email, which covers 5 of 6 features, and no features are covered in the live memory domain (0/7).

FTK, an open source tool, and BEC proprietary software cover all 9 forensic investigation features related to the file-and-disk domain, whereas other tools do not. In the live memory domain system, no available software covers all features of the domain. In the web domain system, the open source FTK covers all its features (6/6), whereas other tools do not. In the email domain system, no software covers all its features.

In the network domain system, no software tool covers all forensic investigation features. In the network domain, IntaForensics (7/8), Nmap (7/8), and LogRhythm (7/8) cover most forensic investigation features, but not all. In the multimedia domain, IntaForensics (6/7) and Cognitech (6/7) proprietary software tools cover most forensic investigation features, but not all.

## 5 Discussion

The findings underscore that open source tools alone cannot comprehensively address all digital forensic investigations. The purpose of digital forensic investigations is to support court proceedings and assist an expert in providing an opinion grounded in logic and evidence within a coherent narrative. However, a challenge in court cases is the admissibility of evidence, which is subject to the Frye and Daubert challenges. The open source tools lack a formalized system of accreditation (Gillett and Fan, 2023).

The Frye challenge requires that the investigation has been conducted using scientifically accepted tools and methods within the scientific community (Court of Appeals of the District of Columbia, 1923). Hence, rather than availability, the scientific acceptability takes precedence in selecting the digital forensic tools.

The Daubert challenge applies to the admissibility of expert opinion and requires demonstrating that expert witness testimony is both relevant and reliable. Meeting relevance must satisfy five elements. (1) The technique has been tested. (2) It has appeared and been discussed in peer-reviewed publications. (3) The potential errors resulting from using tools are known and understood. (4) Tools follow standards of practice. (5) It has widespread acceptance within its scientific community (US Supreme Court, 1993).

The Daubert Challenge has broader applicability in courts: under Rule 702, an expert need not be a scientist; for example, a digital forensic expert may qualify as an expert. These legal proceedings highlight that relying solely on open source digital tools for forensic investigation can be unsatisfactory, and that such evidence can be challenged by the opposing attorney on any or all of the five elements required to satisfy the relevant evidentiary standards. When used as evidence in court proceedings, open source digital tools are challenged because they have likely not undergone formal certification (Gillett and Fan, 2023). It has led to questioning the reliability of forensic evidence generated from these tools (Javed et al., 2022). However, empirical evidence from experimental conditions shows that, when certified, open source digital tools produce results as valid as those of proprietary tools (Ismail and Zainol Ariffin, 2025). Brightening the future of open source tools used in court evidence requires adherence to certification requirements.

Clients and investigators in litigation work with limited resources and specific investigation aims, prioritizing client requirements. Triage can direct investigators to specific domain systems and domain system attributes for forensic investigation, thereby informing the selection of investigative tools (Horsman, 2022). The findings showed that, in one instance, open source tools were the appropriate choice, whereas in other instances, freeware or proprietary tools were the choice. However, the possibility exists of using large language models as adjuncts; such evidence, admission, and acceptance in courts is an evolving field (Dunsin et al., 2024).

## 6 Conclusion

### 6.1 Remarks

The findings showed that the null hypothesis was not supported. The output data from a published journal article served as a case study, demonstrating that open source tools alone are insufficient for conducting a comprehensive forensic investigation and supporting court proceedings (Javed et al., 2022). Their insufficiency can arise from a lack of formal certification (Ismail and Zainol Ariffin, 2025), and the speed with which digital tools can pre-empt and respond to the identification of new artifacts, such as deepfakes. Against this backdrop, forensic investigations are conducted, where the nature, magnitude, and complexity of crimes unfold within an evolutionary landscape. These are additional considerations when selecting the most suitable forensic investigation tools. Additionally, each domain system stores and handles data differently and has distinct forensic investigation attributes. The nature and magnitude of data accepted, processed, and released by computers are constantly evolving across domain systems and forensic investigations, and the attributes of these systems and investigations are also constantly evolving (Kent et al., 2006). It is crucial to consider these factors as digital forensic tools generate evidence for expert witness testimony. These tools assist in generating not merely relevant evidence but rigorous, robust evidence that must be governed by Rule 702; in court, they are presented by forensic experts and contested by forensic experts (Pusey and Ramkissoon, 2026).

Evaluating and contrasting digital forensic tools requires examining domain-specific applications and functional search features, assessing output verifiability and reliability, evaluating ease of use, and assessing the support services provided by the tool manufacturer. Proprietary tool manufacturers are stronger in support services but less likely to share their source code for proprietary reasons (Manson et al., 2007).

The tools investigated in this paper are primarily used in computer forensics. Although it shares common goals with forensic investigation, mobile forensics is substantially different. Mobile forensics involves recovering additional communication data, including images and videos, as well as voice and video calls, by recovering deleted messages and accessing call logs. A vital data repository is the SIM card, which stores contact numbers and text messages. Data are likely backed up in the cloud. Digital forensic tools used are different to meet these unique data extraction requirements. Sleuth Kit, Sans Sift, and Mobiledit are open source tools, while Phone Forensic Expert is a commercial tool. Open source and proprietary tools have comparative strengths; however, proprietary tools tend to offer better speed and accuracy in data extraction and analysis (Padmanabhan et al., 2016). On the other hand, open source tools offer transparent source code that can be presented to courts as auditable evidence of investigations (Carvey and Althede, 2011).

### 6.2 Limitations

The findings of this study are limited by the secondary data used, which were obtained from Javed et al.'s (2022) study. As this paper used secondary data for analysis, the inclusion criteria of those digital tools have influenced the findings. As time passes, new, more refined, and updated versions of open source, firmware, and proprietary tools are released, necessitating revisiting the applicability of the findings to the settings under study.

### 6.3 Future research

Future research that compares digital forensic tools across computers, mobile devices, and the cloud, particularly open and proprietary tools, can provide invaluable insights for researchers, developers, and users. Second, the effectiveness of these tools in various targeted investigations, particularly in the broader areas of fraudulent statements, asset misappropriation, and corruption, remains unexplored. Third, future research can explore the role and extent to which open source tools benefit from large language models (Yin et al., 2025).
